# Changes in Flavor and Volatile Composition of Meat and Meat Products Observed after Exposure to Atmospheric Pressure Cold Plasma (ACP)

**DOI:** 10.3390/foods12173295

**Published:** 2023-09-02

**Authors:** Kathrine H. Bak, Peter Paulsen

**Affiliations:** Institute of Food Safety, Food Technology and Veterinary Public Health, University of Veterinary Medicine Vienna, Veterinärplatz 1, 1210 Vienna, Austria; peter.paulsen@vetmeduni.ac.at

**Keywords:** cold plasma, curing, dielectric barrier discharge, in-package, microplasma, plasma jet, sensory, volatile compounds

## Abstract

Studies on the atmospheric pressure cold plasma (ACP) exposure of meat and meat products mainly determine microbial inactivation, lipid oxidation, and meat color. Some studies include sensory evaluation, but only a few determine the changes in volatile composition due to ACP treatment. The results of sensory evaluation are inconclusive and range from “improvement” to “off-odor”. This could be due to differences in the food matrix, especially in processed foods, or different experimental settings, including inadvertent effects such as sample heating. The few studies analyzing volatile composition report changes in alcohols, esters, aldehydes, and other compounds, but not necessarily changes that are novel for meat and meat products. Most studies do not actually measure the formation of reactive species, although this is needed to determine the exact reactions taking place in the meat during ACP treatment. This is a prerequisite for an adjustment of the plasma conditions to achieve antimicrobial effects without compromising sensory quality. Likewise, such knowledge is necessary to clarify if ACP-exposed meat and products thereof require regulatory approval.

## 1. Introduction

In 1928, Langmuir coined the term *plasma* for the region of an ionized gas near the electrodes, which contains roughly equal numbers of ions and electrons [1]. Plasma is also termed the fourth state of matter, and is, in other words, a conducting gas [2]. Specifically, atmospheric pressure cold plasma (ACP) is plasma generated at atmospheric or reduced pressures, a technology that requires less power input than thermal plasma [3]. ACP is produced by exposing a non-toxic gas to an electric field or to electromagnetic waves [4]. ACP is promising in terms of sustainable food processing [3,4,5], where it has potential as a sanitation technology for food surfaces and food contact materials [6]. Briefly, ACP effectively inactivates microorganisms at low temperatures [3] via reactive species damaging cell membranes, DNA, lipids, and proteins [7,8,9]. Despite the effectiveness of ACP, the technique is not yet readily available for commercial use. As previously discussed by Csadek et al. [7], it is possible that ACP-treated foods may be classified as novel foods according to EU Regulation 2015/2283 [7,10]. The definition of novel foods according to this regulation includes, inter alia, “food with a new or intentionally modified molecular structure, where that structure was not used as, or in, a food within the Union before 15 May 1997” (Article 3, paragraph 2, (i)) of EU Regulation 2015/2283 [10]) or “food resulting from a production process not used for food production within the Union before 15 May 1997, which gives rise to significant changes in the composition or structure of a food, affecting its nutritional value, metabolism or level of undesirable substances” (Article 3, paragraph 2, (i)) [10]. For the application of ACP generated from air, oxidative changes, and reaction of nitrites from the plasma with meat myoglobin have been reported [6], but there is no evidence for the formation of novel structures or compounds or significant quality losses. Food business operators intending to place a particular food on the market in the EU must verify if this is a “novel food”. To this end, the food business operators will have to consult the member state where they first intend to place this food. The member state may contact other member states and the Commission to reach a decision (Article 4 of EU Regulation 2015/2283 [10]). Ultimately, the Commission will decide on the classification of the particular food and its authorization as a novel food. The procedural requirements are laid down in Chapter III of EU Regulation 2015/2283 [10], and will involve an assessment by the European Food Safety Authority (EFSA). In essence, such novel foods must not pose a health risk and must not mislead the consumer. Currently, no ACP-treated foods are included in the EU list of authorized novel foods, Commission Implementing Regulation (EU) 2017/2470 [11], which is periodically updated. The regulatory approval routes in the United States have been reviewed and discussed by Keener and Misra [5] and by Yepez et al. [4]. In summary, due to the complex chemical reactions involved, extensive research must be performed prior to the regulatory approval of ACP as a food technology [5].

Microbial inactivation is the main purpose of ACP application to food with the technology being efficient against bacteria, spores, fungi, and viruses in addition to pesticides and mycotoxins [12]. Hence, the effect of ACP on meat and meat products has mainly been studied in terms of microbial inactivation [7], but it is evident that the formation of reactive oxygen species (ROS) and reactive nitrogen species (RNS) may lead to, e.g., lipid oxidation and color changes [6]. ACP-induced lipid oxidation in meat has been reviewed extensively by, for example, Gavahian et al. [13]. The degree of ACP-induced lipid oxidation depends on numerous factors such as the type of plasma used, gas composition, humidity, and settings such as input power and duration of the ACP treatment. Additional factors include the lipid composition of the food, moisture, storage time post-ACP treatment, and use of antioxidants [13]. It is reasonable to assume that measurable changes in the volatile composition of meat and meat products may occur as a result of ACP, certainly after ACP with oxygen as part of the plasma-generating gas as lipid oxidation forms undesirable volatile compounds [14]. Minor ACP-induced protein oxidation may lead to improved myofibrillar protein functional properties, while severe oxidation should be prevented as it may lead to decreased functional properties [15,16]. Furthermore, ACP can be used for curing meat, either via direct treatment of the meat with ACP or via the use of plasma-treated water (PTW), as reviewed in our previous work [6].

Although numerous papers have been published on the topic of ACP treatment of meat, research on meat quality parameters beyond microbial quality, lipid oxidation, and color is still lacking. As pointed out by Rossow et al. [17], future research should deal with other important meat quality parameters as well. This includes sensory evaluation and determination of volatile composition.

This review provides a brief overview over the most common methods for the generation of ACP for treatment of meat and meat products as well as a description of novel developments in ACP technology. Studies reporting on the effects of ACP treatment on the flavor and volatile composition of meat and meat products are reviewed and discussed. Consequently, gaps in the current knowledge regarding the effect of ACP on parameters beyond microbial quality, lipid oxidation, and meat color are identified, especially as related to flavor.

## 2. Atmospheric Pressure Cold Plasma in Meat Processing

Cold plasma can be generated by a variety of sources: Corona discharge, gliding arc discharge, dielectric barrier discharge (DBD), plasma jet, microwave plasma, inductively coupled plasma, capacitively coupled plasma, and UV photo-ionization [5]. The most common methods for ACP generation in food processing are DBD and plasma jets [18] with DBD seemingly being the most commonly used plasma source when treating meat and meat products [19].

**Figure 1:**
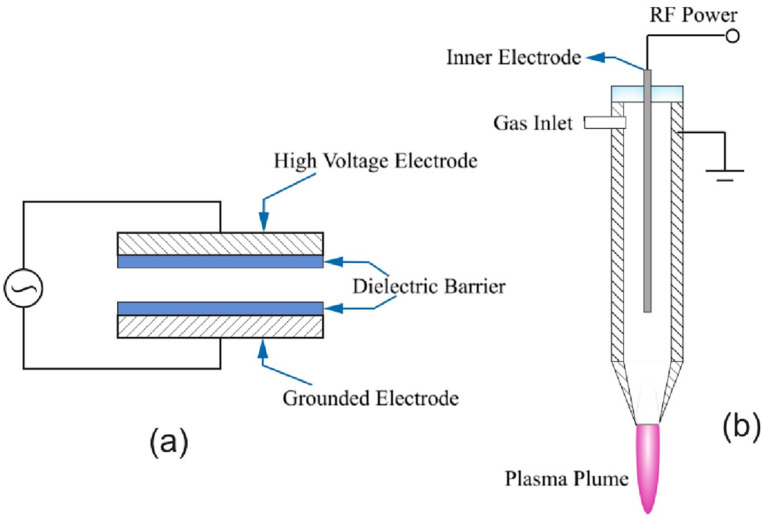
Illustration of (**a**) dielectric barrier discharge and (**b**) plasma jet. Reprinted from [18] with permission from Elsevier.

A DBD plasma generation setup consist of two metal electrodes, at least one coated with a dielectric layer, with a high potential difference applied across them [18] (Figure 1). The distance between the electrodes varies in different studies, and can be up to several cm [20]. Exposing the gas to the electric field at room temperature generates a small percentage of ionized gas, resulting in formation of reactive plasma species such as ROS and RNS, depending on the composition of the treatment gas [4].

The plasma jet can be considered a modification to the other methods for plasma generation [21], though most jets are based on DBD configurations [19]. The plasma jet consists of two concentric electrodes through which the gas or gas mixture flows at a high rate [18,21]. The plasma jet is generally placed a few millimeters above the food, and the ionized gas is directed via the nozzle (Figure 1).

**Figure 2:**
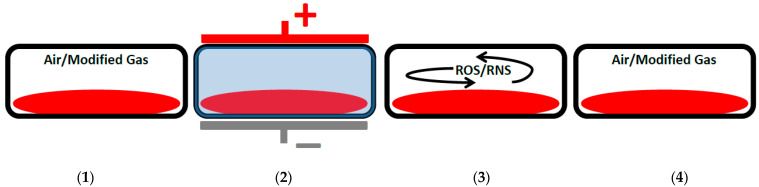
Illustration of the processes taking place during in-package ACP treatment. (**1**) The meat product in a sealed package with air or a modified gas mixture. (**2**) The package is subjected to a high-volage electric field causing gas breakdown and plasma generation. (**3**) The formed reactive oxygen species (ROS) and reactive nitrogen species (RNS) diffuse in the package and inactivate spoilage microorganisms over the span of a few hours. (**4**) The ROS and RNS recombine to recreate the original atmosphere in the package. Inspired by Figure 2 from Misra et al. (2019) [12].

A relatively new concept is the use of in-package DBD [18] and other in-package ACP technologies, which have been reviewed extensively by Misra et al. [12]. In-package ACP works by exposing the already packaged food product to ACP, thereby ionizing the gas in the headspace of the package and causing microbial inactivation in a uniform way. Unreacted plasma-generated species recombine, thus, recreating the original gas in the package (Figure 2) [12]. The future potential of in-package DBD is excellent as it is possible to treat, for example, vacuum-packaged meat products under continuous large-scale conditions [19].

**Figure 3:**
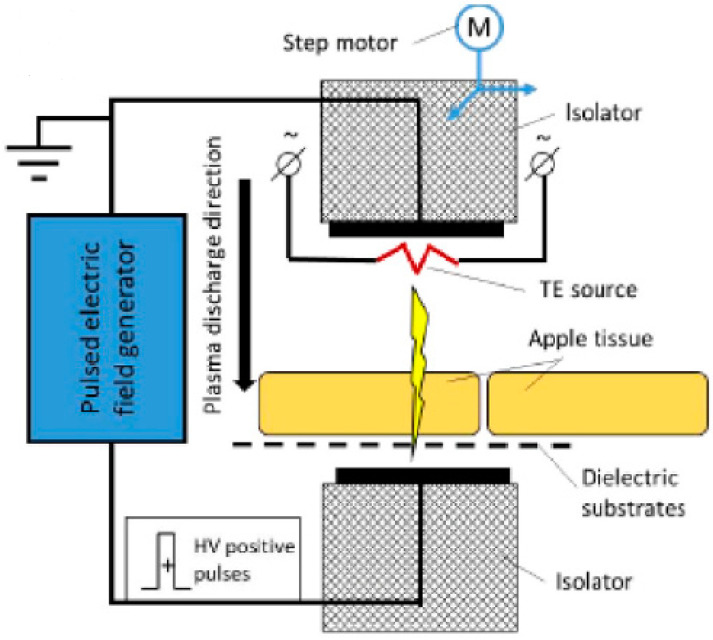
Illustration of cold filamentary microplasma treatment assisted by thermionic emission (TE). Reprinted from [24] with permission from Elsevier.

Another recent development within ACP technology is its use as a drying pretreatment technology, primarily for fruits and vegetables [22]. One such pretreatment ACP method is cold filamentary microplasma (CFM). CFM creates a thin plasma channel by a high voltage, non-self-sustained gas discharge between two metal electrodes in a gaseous medium equipped with permanent magnets to create a concentrated discharge [23,24]. With this technique, it is possible to penetrate the entire food product as opposed to treating only the surface (Figure 3) [24].

CFM as a pretreatment technology has been employed to reduce drying time and improve drying efficiency for plant products, e.g., potato slices [23] and apple slices [24]. Drying is accelerated by the creation of surface micro-holes and electrically induced channels through the material [24], resembling piercing with a needle [23]. It is plausible that this technology might be used on meat products in the future, though this would require extensive studies for optimization of conditions due to the differences in the nature of the matrix such as the higher content of fat and protein in meat. It would be interesting to investigate if CFM could reduce drying time for meat products such as dry-cured hams and jerky without negatively influencing important quality characteristics such as color, texture, and flavor.

## 3. ACP Effect on the Flavor of Meat and Meat Products

Table 1 shows an overview over research studies where the effect of ACP on meat and meat products has been evaluated in terms of a sensory evaluation and/or determination of the volatile composition in the headspace. Since the main task of plasma treatment of foods is to reduce bacteria or viruses on the food surface, some settings use prolonged exposure times to achieve sufficient reductions. Arguably, this bears the risk of heating the sample surface and some of the reported effects might have been caused by a rise in the surface temperature, which could be prevented by cooling systems. With respect to the action of plasma gas species, reduction in exposure times will reduce the risk of unwanted changes, as e.g., lipid oxidation. In order to better understand which reactions take place in the meat matrix, a characterization of the composition of the plasma should be provided [25] in addition to the technical settings (e.g., voltage, frequency, amperage, geometry of the electrodes etc.). Currently, a variety of methods is available for the characterization in air–gas plasmas, depending on if the food item is exposed directly or indirectly (e.g., PTW). In direct treatment settings, analytical methods must be capable of detecting (and quantifying) not only long-lived compounds, but also short-lived ones [26]. A detailed description of available methods and issues of selectivity have been given by Gorbanev et al. [26].

### 3.1. Uncured Meat

ACP-generated ROS can react with unsaturated fatty acids, thus initiating lipid oxidation [25]. Secondary lipid oxidation products, such as many aldehydes, are known to cause off-flavors in meat [14]. Especially when using an O_2_-containing treatment gas, an effect on flavor might be anticipated. Nevertheless, not all studies found ACP to influence flavor (Table 1). For example, no effect was found on the flavor of either raw or cooked pork loin treated with DBD ACP using a gas mixture consisting of He and O_2_ [27]. Similarly, in-package DBD using atmospheric air had no effect on the off-flavor of pork butt and beef loin, though taste was negatively influenced by the treatment for both types of meat [30]. On the other hand, using Corona discharge plasma jet with dried, filtered air as the treatment gas increased the off-flavor of fresh pork slices, while the sensory characteristics of frozen pork slices remained unaffected [31]. A number of studies have dealt with the effect of DBD on chicken breast. A decrease in desirable flavor and an increase in off-flavor with extended in-package DBD exposure time but no effect on taste was found in one study where atmospheric air was the treatment gas [36]. Another study found a decrease in the flavor score of protein-coated boiled chicken breast cubes immediately following in-package DBD treatment, but with no effect between DBD-treated and untreated samples after 3 days of refrigerated storage [38]. DBD on both raw and cooked chicken breast led to improvements in odor and flavor, respectively, after DBD ACP treatment with argon, not surprisingly, performing better than oxygen as the treatment gas [37].

A few studies have employed gas chromatography–ion mobility spectrometry (GC-IMS) as a method to analyze the effect of ACP on the volatile composition of meat. When applying in-package DBD with an Ar-gas to pork meatballs, the content of certain aldehydes, alcohols, and esters increased in the headspace despite the atmosphere being O_2_-free [33]. The aldehydes include nonanal, (E)-2-octenal, (E)-2-nonenal, and benzaldehyde, which have previously been detected in, for instance, reheated pork [39] and stewed goat meat (with the exception of benzaldehyde) [40]. Nonanal has a fruity odor [33,40] and originates from autoxidation of oleic acid or from the degradation of its hydroperoxides or degradation of hydroperoxides from other n-9 monounsaturated fatty acids [39]. (E)-2-octenal has been attributed a grilled meat aroma and is suspected to be derived from linoleic acid degradation [40] or β-scission of n-7 unsaturated fatty acids [39]. (E)-2-nonenal, which has been described as nutty [40] or as having a cooked, cured ham odor [33], originates from β-scission of n-7 unsaturated fatty acids [39]. The odor of benzaldehyde is described as bitter almond [33]. Benzaldehyde may be formed by degradation of the amino acid phenylalanine and its derivatives in the presence of lipid hydroperoxides [41]. The alcohol that saw the most significant increase in concentration due to ACP treatment in the pork meatballs was 2-hexanol [33], originating from the oxidation of polyunsaturated fatty acids [42,43]. The presence of 2-hexanol has previously been described in the volatile composition of, for instance, yak meat [43] and rainbow trout [42], and the odor descriptors are in the areas of green and pungent [33,42]. ACP treatment of pork meatballs also resulted in an increased headspace concentration of a few esters such as hexyl acetate [33], which has a fruity, green odor and has previously been detected in unsmoked bacon [44] and unheated yeast-fermented pork hydrolysate, being one of the main metabolites of the fermentation of the corresponding alcohol [45]. Generally, a larger increase in headspace concentration of volatiles was seen with increasing treatment time for DBD ACP with Ar-gas performed on pork meatballs [33].

When performing in-package DBD ACP with atmospheric air as the treatment gas on fresh beef patties made from longissimus lumborum, it was found that certain alcohols (1-octen-3-ol and 1-pentanol), aldehydes (heptanal, trans-2-heptenal, n-nonanal, hexanal, and octanal), ketones (2,3-pentanedione), and esters (ethyl butanoate and (Z)-3-hexenyl acetate) were produced or increased in concentration [34]. The aroma of the alcohols was overall described as being woody, acrid, grassy, fruity, mushroom, and fatty and produced via a reaction between alkoxyl radicals formed during lipid oxidation and a second lipid molecule [34,46]. The detected aldehydes were described as nutty, fruity green, and fatty (heptanal), fatty and floral (n-nonanal), green, leafy, and grassy (hexanal), and fruity, citrusy, and fatty (octanal), i.e., largely undesirable odors of these secondary lipid oxidation products, which are generally important flavor compounds due to their low odor thresholds [34,39]. The ketone 2,3-pentadione has been correlated positively to the aroma of yak meat [43]. The esters ethyl butanoate and (Z)-3-hexenyl acetate are described as sweet or fruity and originating from esterification of the corresponding alcohols and carboxylic acids [34]. The volatile compounds formed in the beef patties as a result of DBD ACP treatment in air are mainly related to lipid oxidation and could have a negative effect on the eating quality, though no sensory evaluation was conducted to confirm this [34].

### 3.2. Cured Meat and Meat Curing

As reviewed in our previous work, ACP can be used as a way of curing without the addition of nitrite [6] as shown successfully, for example, for pork jerky [47], canned ground ham [29], and a meat batter [48]. Similar to curing with nitrite, curing via ACP largely prevents lipid oxidation, though microbial inactivation may be hampered and the ACP treatment will need to be adjusted accordingly [48]. Sensory characteristics in terms of flavor and taste were found to be either unaffected or improved by curing via DBD ACP instead of traditional curing of canned ground ham [29]. Instead of curing via direct application of ACP to the meat, curing with ACP may also be carried out using PTW with only slightly worse [49] or even slightly improved [50] results in terms of cured color. The important point here is equalization of the nitrite concentration [6].

An extensive study combining the use of the electronic nose, GC-IMS, and sensory evaluation was conducted to assess smoked pork sausage cured either via ACP-treated phosphate-containing brine in combination with (reduced amounts of) nitrite or the traditional way, i.e., via nitrite alone [32]. Interestingly, despite no differences being detected by the sensory panel in terms of smoked flavor and the overall acceptability of the sausages, some differences were detected in the volatile composition, and grouping of the treatments was possible with partial least squares discrimination analysis [32]. For example, sausages with the ACP-treated brine had an increased content of the aldehyde 2-methylbutanal compared to the untreated control and the nitrite-only group [32]. The branched chain aldehyde 2-methylbutanal has previously been positively associated with the flavor development in Jinhua dry-cured ham [51] and detected as a minor odor compound in Iberian dry-cured loin [52]. The compound originates from the Strecker degradation of the amino acid isoleucine [53].

Only one study combines application of ACP treatment of cured meat products with subsequent sensory evaluation. Inactivation of mold and bacteria in beef jerky was investigated and supplemented with a sensory evaluation [35]. Unfortunately, DBD with ambient air as the treatment gas led to slightly decreased scores for flavor along with an increase in off-odor [35]. One might speculate that treatment with an oxygen-free gas could be advantageous in this case, as it is well-known that cured meat is susceptible to the influence of oxygen [54].

It has been shown that DBD ACP can be used to modify myofibrillar protein structure and, therefore, their ability to bind volatile flavor compounds [28]. Myofibrillar proteins extracted from dry-cured bacon treated with DBD ACP saw a decrease in five different aldehydes in the headspace, especially with ACP treatment at 70 kV, but also at 60 kV compared to 50 kV and the untreated control (Table 1). The authors suggest DBD ACP as a method for modifying the functionality of myofibrillar proteins while improving the flavor of meat products [28]. Optimizing ACP conditions in order to obtain desirable results for all quality parameters is important. In addition, as shown by Luo et al. [28], increasing the voltage may actually reduce the headspace concentration of certain undesirable aldehydes.

### 3.3. Research Gaps

Comparably few research data have been published on the effects of ACP treatment of meat and meat products on meat quality parameters beyond microbial quality, lipid oxidation, and color.

It is well-known that ACP treatment can affect the activity of endogenous food enzymes [18,55]. However, the effect of ACP on metmyoglobin reductase, which is critical for maintaining color stability in fresh meat, has not yet been investigated [6], though this might help explain observed color changes following ACP treatment. As myoglobin oxidation is known to accelerate lipid oxidation (and vice versa) [56], any ACP-induced reduction in color stability could lead to an increase in lipid oxidation, and, consequently, off-flavor development. Generally, it can be concluded that ACP decreases enzyme activity due to ROS and RNS destroying chemical bonds within the enzymes, leading to folding and conformational changes causing a reduction in enzyme activity [18,22].

Another significant research gap is the sensory changes taking place because of ACP. Though some studies do include a sensory evaluation, very few studies further include analysis of changes in volatile composition due to ACP treatment. This could certainly be advantageous when trying to explain any sensory changes taking place. Many studies speculate on the reactive species formed due to ACP, which are responsible for the observed changes in quality parameters. Nonetheless, most studies do not actually measure this. One exception is Rød et al. [57], who measured the ozone concentration after DBD ACP treatment of bresaola with 70% Ar and 30% O_2_ as the treatment gas, but not the concentrations of other reactive species. Knowledge of reactive species formed and changes in volatile composition is necessary to determine the exact reactions taking place resulting in ACP-induced changes in flavor as well as other meat quality parameters. This information will make it easier to adjust the plasma conditions to achieve the desired outcome.

## 4. Conclusions

Studies on the effect of ACP treatment on meat and meat products mainly determine microbial inactivation, lipid oxidation, and meat color. Some studies include sensory evaluations, but only a few determine the changes in volatile composition due to ACP treatment. Most studies lack a thorough characterization of the reactive species formed due to ACP, which effectuate changes in flavor and other quality parameters. Such knowledge would not only allow fine-tuning of ACP treatment in terms of minimum sensory alteration but will also be needed to determine if ACP-treated foods must be considered as “novel foods” requiring regulatory approval.

## Figures and Tables

**Table 1:** Effect of atmospheric pressure cold plasma (ACP) treatment on meat and meat products in terms of flavor and volatile composition.

	Meat Type	Plasma Source	Treatment Variables	Method of Analysis	Results	Reference
Pork	Loin (raw and cooked)	DBD ^a^	He + O_2_ 3 kV; 30 kHz 5 or 10 min	Sensory evaluation	No effect on flavor, odor, and acceptability	[27]
	Myofibrillar protein from dry-cured bacon	DBD	Ambient air 50, 60, or 70 kV 150 s	Binding of aroma compounds	Increased binding of the five investigated aldehydes at 60 and 70 kV	[28]
	Canned ground ham cured with ACP or NaNO_2_	DBD	Ambient air 7 kV; 25 kHz 30 min	Sensory evaluation	No effect on flavor Improved taste and overall acceptability compared to NaNO_2_ addition	[29]
	Butt	In-package DBD	Atmospheric gas 15 kHZ; 100 W peak power, 2 W average power 2.5, 5, 7.5, or 10 min	Sensory evaluation	Negative effect on taste No effect on off-flavor and overall acceptability	[30]
	Frozen and fresh ^b^	CDPJ ^c^	Dried, filtered air 20 kV; 58 kHz 30, 60, 90, or 120 s	Sensory evaluation	No changes for frozen pork Negative effect on off-flavor and acceptability, increasing with increasing treatment time for fresh pork	[31]
	Smoked sausage cured with ACP-treated brine in combination with NaNO_2_ or NaNO_2_ alone	CP discharge	40 W 45 min	Electronic nose GC-IMS ^d^ Sensory evaluation	Increased response value for sensors W1W (sulfur-organic), W2W (sulfur-chlorine), W5S (broad range), W1S (broad-methane), W2S (broad-alcohol), and W3S (methane-aliphatic) with CP-treatment; decreased response for sensors W1C (aromatic), W3C (aromatic), and W5C (aromatic-aliphatic) Possible to distinguish treatment groups based on volatile composition No significant differences in flavor	[32]
	Meatballs	In-package DBD	Ar 85 kV 3, 6, or 9 min	GC-IMS	Increase in certain aldehydes, alcohols, and esters	[33]
Beef	Fresh *longissimus lumborum* patties	In-package DBD	Air 70 kV; 50 Hz 10 min	GC-IMS	Specific alcohols, aldehydes, ketones, and esters detected	[34]
	Loin	In-package DBD	Atmospheric gas 15 kHz; 100 W peak power, 2 W average power 2.5, 5, 7.5, or 10 min	Sensory evaluation	Negative effect on taste No effect on off-flavor and overall acceptability	[30]
	Jerky	In-package DBD	Ambient air 15 kHz 2.5, 5, or 10 min	Sensory evaluation	ACP for 10 min slightly decreased scores for flavor and acceptability and increased off-odor	[35]
Poultry	Chicken breast	In-package DBD	Atmospheric air 15 kHz; 100 W peak power, 2 W average power 2.5, 5, 7.5, or 10 min	Sensory evaluation	Decrease in flavor and increase in off-flavor with extended ACP exposure time No effect on taste and acceptability	[36]
	Chicken breast fillet	DBD	O_2_ or Ar 20 kV, 200 MHz 3 or 5 min	Sensory evaluation	Improved sensory scores in both raw (odor and overall acceptability) and cooked (flavor and overall acceptability) Ar better than O_2_	[37]
	Protein-coated boiled chicken breast cubes	In-package DBD	Max. 40 kV, 60 Hz 2, 2.5, or 3 min	Sensory evaluation	Decrease in flavor score immediately after ACP treatment, no difference after 3 days of storage at 4 °C	[38]

^a^ Dielectric barrier discharge (DBD). ^b^ No specific muscle mentioned. ^c^ Corona discharge plasma jet (CDPJ). ^d^ Gas chromatography–ion mobility spectrometry (GC-IMS).

## Data Availability

Not applicable.
